# 3D-Printed Soft Bionic Inchworm Robot Powered by Magnetic Force

**DOI:** 10.3390/biomimetics10040202

**Published:** 2025-03-26

**Authors:** Deli Xia, Luying Zhang, Weihang Nong, Qingshan Duan, Jiang Ding

**Affiliations:** 1 Guangxi Key Laboratory of Manufacturing System and Advanced Manufacturing Technology, School of Mechanical Engineering, Guangxi University, Nanning 530004, China; 2211301072@st.gxu.edu.cn (D.X.); 2311301081@st.gxu.edu.cn (L.Z.); 2411401173@st.gxu.edu.cn (W.N.); 2 School of Light Industry and Food Engineering, Guangxi University, Nanning 530004, China; qs_duan@gxu.edu.cn; 3 State Key Laboratory of Featured Metal Materials and Life-Cycle Safety for Composite Structures, Guangxi University, Nanning 530004, China

**Keywords:** bionic inchworm, soft robot, magnetic force, 3D-printed

## Abstract

Based on soft body structure and unique gait of bending and stretching, Soft Bionic Inchworm Robots (SBIRs) are used in pipeline inspection and terrain exploration. Many existing SBIRs rely on complex production mechanisms and are cable-driven, which hinders rapid production and smooth movement through complex environments, respectively. To address these challenges, this paper introduces a 3D-printed SBIR, featuring a 3D-printed body actuated by magnetic forces. We introduce the design and production process of the 3D-SBIR and analyze its motion gait. Subsequently, the material composition model and bending deformation model of the robot are developed based on the theory of hyper-elastic materials. The accuracy of the model is validated using simulation analysis and experimental testing of the robot. Meanwhile, we carry out a magnetic simulation analysis and discuss the factors influencing the size of the magnetic force. Finally, a series of experiments are conducted to prove the excellent locomotion capability of the robot. The 3D-SBIR demonstrates remarkable flexibility and multimodal movement capabilities. It can navigate through narrow curved passages with ease, passively overcome obstacles, climb steps up to 0.8 times its body height, and perform a seamless transition while moving across a horizontal plane onto a vertical plane. The 3D-SBIR proposed in this paper is characterized by rapid production, cable-free actuation, and multimodal motion capabilities, making it well suited for moving in unstructured environments.

## 1. Introduction

Soft bionic robots inspired by soft-bodied animals in nature have become a popular research topic because of their potential to perform various specialized tasks in complex environments, such as space exploration, biomedical exploration, and more [1,2,3]. The inchworm, a common mollusk, relies on the coordination between a flexible body and alternately moving legs to achieve a special gait of bending and stretching. Inspired by the soft body structure and distinct gait of the inchworm, the soft bionic inchworm robot (SBIR) adapts well to environmental changes and shows excellent adaptability to unstructured environments [4,5,6].

However, most existing SBIRs are driven by voltage actuation [7,8,9], fluid actuation [10,11,12], or shape memory alloy actuation [13]. These actuation methods require external electric wires or pneumatic pipes, which will hinder the movement of the robot in special environments. Meanwhile, most of the existing SBIRs are tedious to produce and require more assembly processes. Conversely, cable-free actuation, exemplified by magnetic actuation, can liberate the robot from the bondage of cables. In certain special scenarios, such as conducting inspections within semi-enclosed pipes or delivering medications inside animal bodies, this actuation allows the robot to execute tasks more effectively [14,15,16,17,18]. Rapid production technologies, exemplified by 3D-printed technology, are capable of rapidly translating researchers’ design concepts into physical models. This allows for immediate testing and analysis to identify potential design flaws. Consequently, it effectively shortens the research and development cycle, reduces research and development costs, and expeditiously facilitates rapid design iterations [19,20,21]. Some researchers also combine the advantages of magnetic actuation and 3D-printed technology to develop new SBIRs. As an example, Erina et al. combine 3D-printed technology and magnetic actuation to present a fully 3D-printed, inchworm-like soft robot that is magnetically actuated to move and crawl on the horizontal plane [22]. While the robot achieves cable-free actuation and rapid production, it loses capability for multimodal movement and only moves on a plane surface. It is noteworthy that current SBIRs primarily focus on pipe-climbing or horizontal surface locomotion. Significant challenges remain when these robots attempt to climb over obstacles or execute transitional movements from horizontal to vertical surfaces.

In this study, a 3D-printed soft bionic inchworm robot (3D-SBIR) powered by magnetic force is designed and fabricated, achieving rapid production, cable-free actuation, and multimodal movement capability. The flexible body of the robot is produced in one single step by 3D-printed, eliminating the necessity for a complex production process. By embedding magnets in the flexible body, a non-contact, cable-free power input is provided to the robot. Furthermore, the robot exhibits multimodal movement capabilities, including climbing obstacles and the ability to climb from horizontal to vertical surfaces. A systematic comparison, as shown in Table 1, of the 3D-SBIR with previous inchworm-like robots demonstrates the spatial mobility of the proposed 3D-SBIR design.

Similarly to the natural inchworm, the robot moves by coordination between the bending deformation of its belly and the alternate motion of its legs. This research focuses on analyzing the robot’s gait and force conditions as it climbs an obstacle. Based on the theory of hyper-elastic materials [23,24,25], the constitutive model of the material and a bending deformation model of the robot are established. Finite element analysis and experiments of the bending deformation process of the robot are conducted to validate the material constitutive model and the bending deformation model. Finally, a series of experiments are performed to evaluate the movement performance of the 3D-SBIR.

## 2. The Design and Production Process of the 3D-SBIR

**Figure 1:**
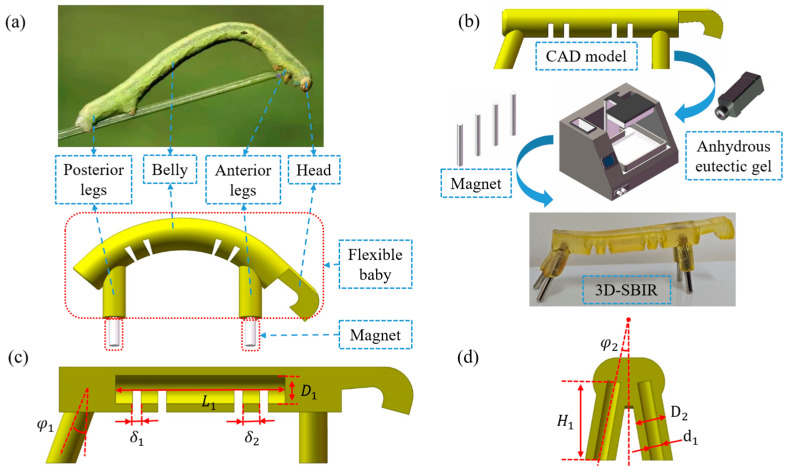
3D-SBIR design and production process: (**a**) Structure comparison between the robot and an inchworm. (**b**) The production process of the flexible body. (**c**) Cutaway view of the robot’s flexible body. (**d**) Cutaway view of robot’s anterior legs.

As shown in Figure 1a, the 3D-SBIR consists of a flexible body capable of bending in multiple directions and four neodymium-iron-boron (NdFeB) magnets. The flexible body is designed to imitate the body structure of the inchworm, with a head, belly, anterior legs, and posterior legs. The belly undergoes bending or stretching deformation under the combined action of the anterior and the posterior legs. Both the anterior and posterior legs feature gaps to embed NdFeB magnets serving as the power source of the 3D-SBIR. As shown in Figure 1b, the flexible body is made of anhydrous eutectic gel, providing excellent flexibility. The body is directly formed by printing with light-curing printing technology. Its adhesion after printing enables four NdFeB magnets to be embedded directly into the gap of the four legs without using additional glue. After inlaying the NdFeB magnets into the gaps, the flexible body undergoes drying to minimize surface adhesion. The 3D-SBIR measures 60 mm in total length, 12 mm in width, and 20 mm in height. Four NdFeB magnets are cylindrical, each measuring 15 mm in length and 2.0 mm in diameter.

Figure 1c provides a cutaway view of the flexible body of the 3D-SBIR, sliced along the axial midsection of the belly. The robot relies on the coordination of the alternating movement of the legs and the bending deformation of the belly to achieve a gait of bending and stretching while moving. The belly is a hollow structure with four gaps at the bottom to allow for better bending deformation. Figure 1d shows a cutaway view of the anterior legs of the 3D-SBIR, with the anterior legs tilted outward relative to the centerline of the belly. The posterior legs are similar to the anterior legs, tilted outward and backward relative to the centerline of the belly and the anterior legs, respectively, enhancing the stability of the robot during movement. The design parameters of the flexible body are outlined in Table 2.

## 3. The Motion Gait Analysis of the 3D-SBIR

### 3.1. Gait Analysis of Motion on a Horizontal Plane

**Figure 2:**
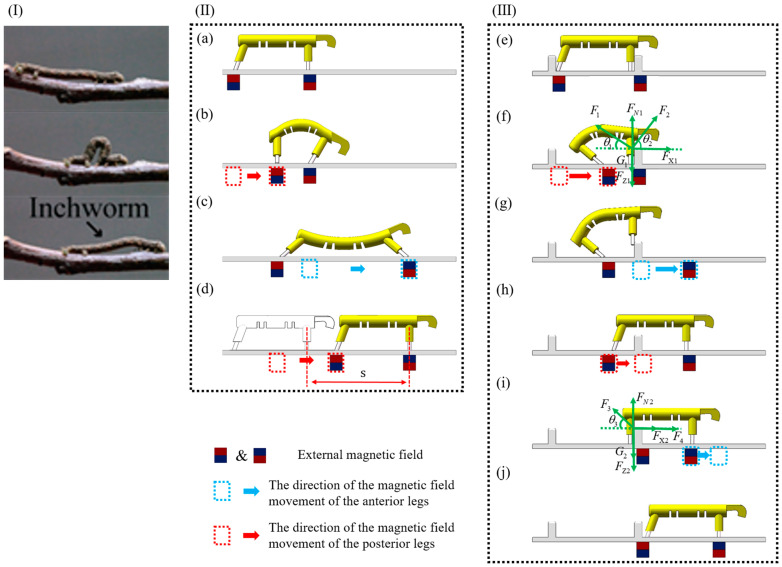
Motion gait diagram of the 3D-SBIR: (**I**) The motion gait of the inchworm [16]. (**II**) Gait diagram of motion on a horizontal plane: (**a**) Initial state of the robot moving on the horizontal plane. (**b**) Bending state of the robot moving on the horizontal plane. (**c**) Stretching state of the robot moving on the horizontal plane. (**d**) Natural state of the robot moving on the horizontal plane. (**III**) Gait diagram of climbing obstacles: (**e**) Initial state of the robot climbing obstacles. (**f**) State of potential energy accumulation for the robot climbing obstacles. (**g**) State of potential energy release for the robot climbing obstacles. (**h**) Moving state of the robot climbing obstacles. (**i**) Passive deformation state of the robot climbing obstacles. (**j**) Natural state of the robot climbing obstacles.

When the 3D-SBIR moves on a horizontal plane, it achieves a bending and stretching motion gait by utilizing the alternating movement of the legs and the bending deformation of the belly. As shown in Figure 2I, Similarly to its biological counterpart in nature, the robot replicates the concertina-style gait characterized by alternating bending and stretching motions. However, unlike the natural inchworm, which relies on synchronized muscle contractions, the 3D-SBIR accomplishes a full locomotion cycle through a four-step actuation sequence. As shown in Figure 2a, in the initial state of the 3D-SBIR moving on the horizontal plane, the robot has vertical anterior legs and posterior legs tilted backward, which ensures the stability of the robot. As shown in Figure 2b, the magnetic field of the anterior legs remains stationary, whereas the magnetic field of the posterior legs moves to the right, forcing the belly to undergo a bending deformation. The posterior legs gradually change from a backward-tilted state to a vertical state, while the anterior legs are tilted forward under the influence of the bending deformation of the belly. As shown in Figure 2c, the magnetic field of the anterior legs moves to the right, whereas the magnetic field of the posterior legs remains stationary, causing the belly to undergo a stretching deformation. The anterior legs gradually change from a forward-tilted state to a backward-tilted state, while the posterior legs are tilted forward under the influence of the stretching deformation of the belly. As shown in Figure 2d, the magnetic field of the anterior legs remains stationary, and the magnetic field of the posterior legs moves to the right. The belly returns to the natural state, and the robot advances a distance of s to the right.

### 3.2. Gait Analysis of Climbing Obstacles

Research on rigid robots when climbing obstacles often prevents direct contact or collision between robots and obstacles to avoid damage to environments or injury to robots. However, the 3D-SBIR, with its soft body structure, is highly adapted to different environments and minimizes environmental damages. This study makes use of this feature to assist the robot in climbing obstacles quickly, leveraging direct contact between the robot and the obstacle as well as contact deformation of the robot while climbing the obstacle.

To better explain the advantages of the 3D-SBIR in climbing obstacles, we analyze the force conditions when the robot climbs obstacles. As shown in Figure 2e, in the initial state of the 3D-SBIR climbing obstacles, there are obstacles on both anterior and posterior legs of the robot. As shown in Figure 2f, in the state of potential energy accumulation for the 3D-SBIR climbing obstacles, when the magnetic field of the anterior legs remains stationary and the magnetic field of the posterior legs moves forward, forcing the belly to undergo bending deformation and accumulate elastic potential energy. In this moment, the anterior legs are affected by a combination of five forces: $G_{1}$ (the force of gravity on the anterior legs), $F_{t1}$ (the force of the attraction of magnetic fields to the anterior legs, which can be decomposed into the force $F_{X1}$ in the horizontal direction and the force $F_{Z1}$ in the vertical direction), $F_{N1}$ (the force of the environmental support for the anterior legs), $F_{1}$ (the force of the obstacle on the anterior legs), and $F_{2}$ (the force of the belly on the anterior legs). The forces on the anterior legs satisfy the following force balance equation:

(1) $$F_{X1}+F_{2}\mathrm{Cos}(\theta_{2})=F_{1}\mathrm{Cos}(\theta_{1})$$

(2) $$F_{N1}+F_{1}\mathrm{Sin}\left(\theta_{1}\right)+F_{2}\mathrm{Sin}\left(\theta_{2}\right)=G_{1}+F_{Z1}$$

Figure 2g shows the state of potential energy release for 3D-SBIR during obstacle climbing. The magnetic field of the anterior legs moves forward while that of the posterior legs remains stationary, resulting in a gradual decrement in the vertical magnetic attraction force on the anterior legs ($F_{Z1}$) and a concurrent increase in the horizontal attraction force ($F_{X1}$). This consequently leads to an enhanced reaction force from the obstacle on the anterior legs ($F_{1}$), whereas the belly force acting on the anterior legs remains constant. These combined effects ultimately cause the environmental support force on the anterior legs ($F_{N1}$) to decrease until it reaches zero. At this critical juncture, the anterior legs are raised upward, the belly releases its stored elastic potential energy, and the robot successfully climbs over the obstacle with its anterior legs. Table 3 lists the trends of each force change during anterior legs obstacle climbing.

As shown in Figure 2h, in the moving state of the 3D-SBIR climbing obstacles, the magnetic field of the anterior legs remains stationary, and the magnetic field of the posterior legs moves until the posterior legs reach the front of the obstacle. Figure 2i shows the passive deformation state of the 3D-SBIR climbing obstacles. When the magnetic field of the anterior legs continues to move forward, the motion of the posterior legs is obstructed, and the belly is forced to undergo stretching deformation. In this moment, the posterior legs are affected by a combination of five forces: $G_{2}$ (the force of gravity on the posterior legs), $F_{t2}$ (the force of the attraction of magnetic fields to the posterior legs, which can be decomposed into the force $F_{X2}$ in the horizontal direction and the force $F_{Z2}$ in the vertical direction), $F_{N2}$ (the force of the environmental support for the posterior legs), $F_{3}$ (the force of the obstacle on the posterior legs), and $F_{4}$ (the force of the belly on the posterior legs). The forces on the posterior legs satisfy the following force balance equation:

(3) $$F_{4}+F_{X2}=F_{3}\mathrm{Cos}\left(\theta_{3}\right)$$

(4) $$F_{N2}+F_{3}\mathrm{Sin}\left(\theta_{3}\right)=G_{2}+F_{Z2}$$

As the magnetic field of the anterior legs continues to move forward, the force of the belly on the posterior legs ($F_{4}$) gradually increases while the force of the attraction of magnetic fields to the posterior legs ($F_{t2}$) remains constant, resulting in a gradual increase in the force of the obstacle on the posterior legs ($F_{3}$). As the force of the obstacle on the posterior legs ($F_{3}$) gradually increases, the force of gravity on the posterior legs ($G_{2}$) remains unchanged, resulting in a gradual decrease in the force of the environmental support on the posterior legs ($F_{N2}$), until this force reaches zero. Figure 2j shows the natural state of the 3D-SBIR while climbing obstacles. The belly releases elastic potential energy, and the posterior legs of the robot climb over the obstacle. The trend of each force change when climbing obstacles with the posterior legs is shown in Table 3.

## 4. Model Construction and Simulation Analysis

### 4.1. Constitutive Model of the Materials

**Figure 3:**
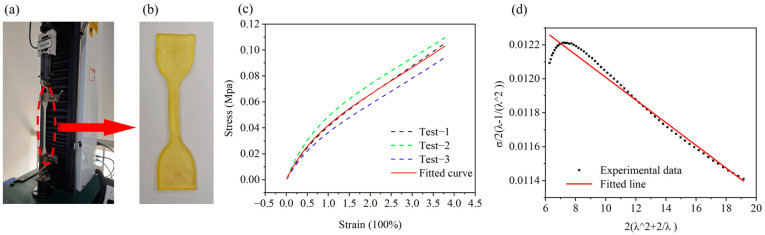
Testing of the material in uniaxial tension: (**a**) Material is stretched on a universal testing machine. (**b**) Standard sample of the material. (**c**) Stress–strain curve of the material. (**d**) Linear fitting of the material constants to the Yeoh model.

The body of the 3D-SBIR is an anhydrous eutectic gel, which provides the same hyperelasticity as silicone rubber. The constitutive model of the material and the bending deformation model of the robot are developed based on the theory of hyperelastic materials. The mechanical behavior of silicone rubber-based hyperelastic materials is generally described using two approaches. One approach establishes stress–strain relationships based on microscopic statistical mechanics, such as the Neo-Hookean model. The other approach establishes stress–strain relationships based on continuum phenomenological theory, including models such as the Ogden model, the Mooney-Rivlin model, and the Yeoh model. The Yeoh model, primarily applied to the large deformation behavior of carbon black filling, requires only simple data from uniaxial tensile experiments to simulate the deformation results well and is suitable for large deformation conditions. The relationship between main stress $\sigma_{1}$ and main tensile ratio $\lambda_{1}$ is established based on the Yeoh model as follows (The detailed procedure of derivation is given in S1):

(5) $$\frac{\sigma_{1}}{2\left(\lambda_{1}-\frac{1}{\lambda_{1}^{2}}\right)}=2C_{20}\left(\lambda_{1}^{2}+\frac{2}{\lambda_{1}}\right)+C_{10}-6C_{20}$$

where $C_{10}$ and $C_{20}$ are the material constants of the Yeoh model, measured from uniaxial tensile tests of the material. As shown in Figure 3a, we conduct uniaxial tensile tests of the material, the thickness of the specimen is 3 mm.

The displacement and load data are obtained from uniaxial tensile experiments on the material. Corresponding data within the displacement range of 0~1 mm is averaged. Similarly, data with displacements of 1~2 mm, 2~3 mm, 3~4 mm and so on are processed in the same manner to calculate stress and strain values. Three sets of tests are conducted, and the stress–strain curve of the material was fitted, as shown in Figure 3c. To determine the material constants $C_{10}$ and $C_{20}$, let $2\left(\lambda_{1}^{2}+2/\lambda_{1}\right)$ and $\sigma_{1}/\left[2\left(\lambda_{1}-1/\lambda_{1}^{2}\right)\right]$ represent the horizontal and vertical axes of the coordinate system, respectively. Considering that the uniaxial tensile test exhibits a significant error in the initial loading of several sets of data, the data within 20% of the strain are removed. Considering that the 3D-SBIR is working with a strain of 200% or less, the data sets where the strain is larger than 200% are excluded. As shown in Figure 3d, the fitted straight line of material constants for the Yeoh model is obtained, $C_{10}=0.0123$, $C_{20}=-6.68e-5$.

### 4.2. The Bending Deformation Model of the 3D-SBIR

**Figure 4:**
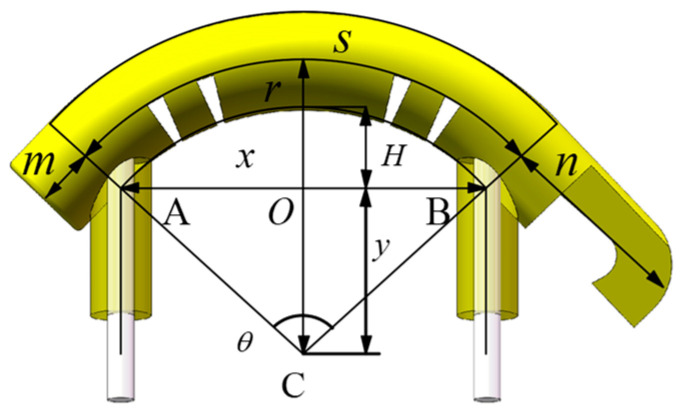
The diagram of the bending deformation of the 3D-SBIR.

When the 3D-SBIR is bent and deformed by an external magnetic field, it is divided into a deformed part and a non-deformed part (no stretching and compressive deformation of the m and n parts). The belly of the 3D-SBIR undergoes bending deformation under the action of external forces. Assuming that the middle layer of the belly does not undergo tensile deformation and serves as a neutral layer, the uppermost layer of the belly undergoes tensile deformation, while the lowermost layer of the belly undergoes compressive deformation. Assuming that the belly undergoes bending by equal curvature, as shown in Figure 4, let r is the radius of curvature of the neutral layer, s is the curvature length of the neutral layer, θ is the center angle of the circle of the neutral layer, the distance of OC is $L_{OC}=y$, the height of the belly lifting is H, and the distance between the anterior and posterior legs is $L_{AB}=x$. If the neutral layer does not undergo tensile deformation, s=40, then there is,

(6) $$\frac{2\pi r\theta }{360}=s,$$

(7) H=r−4−y.

According to the law of sines for ΔABC and ΔAOC, it can be obtained,

(8) $$\frac{x}{\mathrm{Sin}\theta }=\frac{r-4}{\mathrm{Sin}\left(90-\theta /2\right)},$$

(9) $$\frac{r-4}{\mathrm{Sin}\left(90\right)}=\frac{y}{\mathrm{Sin}\left(90-\theta /2\right)}.$$

From Equations (6) and (8), it can be obtained,

(10) $$\frac{x}{\mathrm{Sin}\left(\frac{7200}{\pi r}\right)}=\frac{r-4}{\mathrm{Sin}\left(90-\frac{3600}{\pi r}\right)}.$$

Therefore, we can conclude that r is a function of x, denoted as,

(11) $$r=r\left(x\right).$$

From Equations (6) and (9), it can be obtained,

(12) $$y=\left(r-4\right)\mathrm{Sin}\left(90-\frac{3600}{\pi r}\right).$$

Therefore, we can conclude that y is a function of r, denoted as,

(13) $$y=y\left(x\right).$$

From Equations (7), (11) and (13), the relationship between the height of the belly lift (H) and the distance between the anterior and posterior legs (x) is obtained,

(14) $$H=r\left(x\right)-4-y\left(x\right).$$

### 4.3. Simulation and Experimentation of the Bending Deformation

To verify the correctness of the material constitutive model and the bending deformation model of the 3D-SBIR, and to more intuitively demonstrate the bending deformation process of the robot under the action of external force, finite element analysis of the robot is carried out based on Abaqus. The 3D model is directly established in Abaqus, with a hyperelastic material assigned to the body and the Yeoh model selected for the strain energy density function. Steel material is assigned to the NdFeB magnet. The NdFeB magnet interacts with the flexible body, and its deformation is negligible compared to the deformation of the flexible body. Rigid body constraints are applied to the NdFeB magnet to simplify the simulation process. With the anterior legs’ magnet completely fixed, a body force is applied to the posterior legs’ magnet, moving it 20 mm forward.

**Figure 5:**
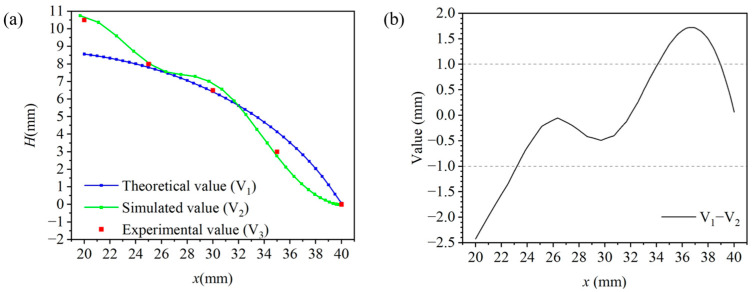
Simulation and experiment of the 3D-SBIR bending deformation: (**a**) Theoretical, simulated and experimental values during bending deformation of the robot (**b**) Difference between theoretical and experimental values.

We conducted an experiment on the bending deformation of the 3D-SBIR to verify the accuracy of the above results of bending deformation simulation. By applying external force directly to the robot, with the magnets of the anterior legs remaining stationary and the magnets of the posterior legs moving forward, we observed the relationship between the height of the belly lift (H) and the distance between the anterior legs and the posterior legs (x). Figure 5a shows the theoretical, simulated, and experimental values of the bending deformation process of the robot. Simulation values closely match the experimental data, demonstrating the accuracy of simulation results, and confirming the validity of the material constitutive model developed using the Yeoh model. Figure 5b shows the difference between the theoretical and simulated values. When the distance x is between 23 mm and 34 mm, the difference is within 1 mm and the bending deformation model is applicable to the robot. When x > 34 mm, the abdomen primarily undergoes compressive de-formation, making the bending deformation model inapplicable. When x < 23 mm, there is not only a bending deformation of the abdomen, but also an upward slip, so the bending deformation model is not applicable in this range either.

### 4.4. Magnetic Simulation and Analysis

**Figure 6:**
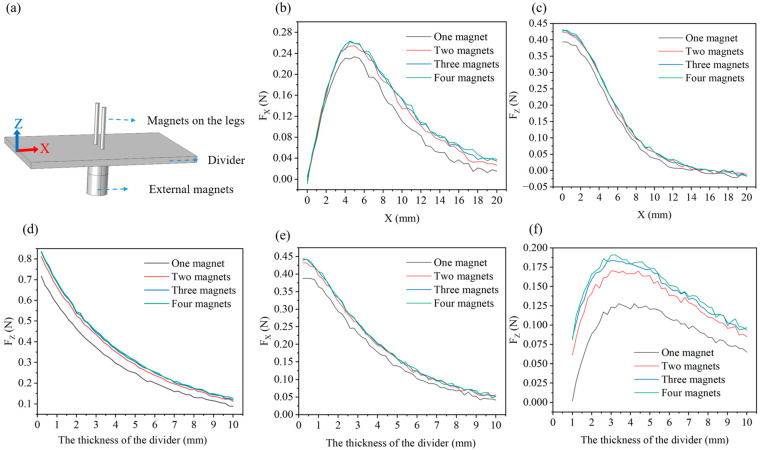
Simulation analysis of the magnetic forces: (**a**) Model for the magnetic forces simulation analysis. (**b**) Relationship between the magnetic forces in the horizontal direction ($F_{X}$ ) and forward displacement of the bottom magnets (X). (**c**) Relationship between the magnetic forces in the vertical direction ($F_{Z}$) and forward displacement of the bottom magnets (X). (**d**) Relationship between the magnetic forces in the vertical direction ($F_{Z}$) and the thickness of the divider. (**e**) Relationship between the magnetic forces in the horizontal direction ($F_{X}$) and the thickness of the divider when the bottom magnets are horizontally offset by 5 mm. (**f**) Relationship between the magnetic forces in the vertical direction ($F_{Z}$) and the thickness of the divider when the bottom magnets are horizontally offset by 5 mm.

The motion of the robot is dependent on the magnetic force provided by external magnets, so we discuss the main factors affecting the size of magnetic force. We perform magnetic simulation of the robot based on Comsol. The 3D model is built directly in Comsol, as shown in Figure 6a. A magnetically insulated simulation space is set up, along with a divider having magnetic permeability of one. The two magnets on the divider represent the two magnets on the anterior or posterior legs, while the two magnets beneath the divider represent the magnets that provide the external magnetic field to the anterior or posterior legs. We analyze the influence of different numbers of external magnets and varying divider thicknesses on the magnetic force exerted by the magnets.

The top two magnets are maintained in a stationary position while the bottom magnets are permitted to translate along the *X*-axis to investigate the relationships between the *X*-direction magnetic forces ($F_{X}$) and displacement (X), as well as between the *Z*-direction magnetic forces ($F_{Z}$) and displacement (X). The corresponding results are illustrated in Figure 6b,c. The results show that the $F_{X}$ reaches its maximum when the moving distance is between 4 and 5 mm, while the $F_{Z}$ keeps decreasing. Additionally, both $F_{X}$ and $F_{Z}$ increase with the number of external magnets, and using two magnets is the most suitable solution. Next, we keep magnets stationary and vary the thickness of the divider to obtain the relationship between the $F_{Z}$ and the thickness of the divider, as shown in Figure 6d. The results reveal that the $F_{Z}$ decreases gradually as the divider thickness increases and increases with the number of external magnets. Furthermore, we offset the top and bottom magnets by 5 mm and vary the thickness of the divider to examine the relationship between the $F_{X}$ and the divider thickness, as shown in Figure 6e. The results are similar to those of Figure 6d, where the $F_{X}$ decreases with increasing thickness of the divider and increases with the number of magnets. Unlike, as shown in Figure 6f, the $F_{Z}$ is increasing and then decreasing with the thickness of the divider. Combining the above magnetic simulation results, we conclude that the best performance is achieved with two external magnets, providing a magnetic force of at least 0.16 N in the horizontal direction and 0.29 N in the vertical direction when the divider thickness is 5 mm or less. (The robot weighs only 5.9 g.)

**Figure 7:**
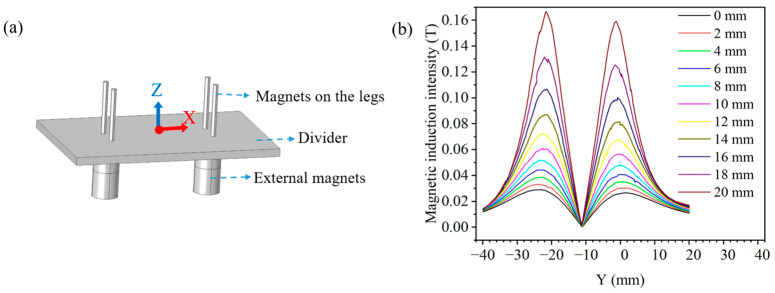
Simulation analysis of magnetic induction intensity: (**a**) Model of magnetic induction intensity simulation. (**b**) Variation in magnetic inductive on the *Z*-axis.

Meanwhile, we investigate the variation in the magnetic induction intensity produced by the magnets as it moves. The magnetic induction intensity simulation is modeled as shown in Figure 7a. Initially, the external front and rear magnets are spaced 40 mm away from each other. We keep the external magnets in front stationary so that the external magnets at the rear moves forward (D mm). Create a moving axis (Z), which is always centered between the front and rear external magnets. The zero scale of the *Z*-axis is in the middle of the divider, oriented vertically upward. We obtain the variation in magnetic induction intensity at each point on the *Z*-axis for every D mm of forward movement of the rear magnet, as shown in Figure 7b. As D increases and the two external magnets continue to move closer together, the magnetic induction intensity at points on the *Z*-axis will gradually increase. We observe that the magnetic induction intensity appears to peak at Z=−21.5 and Z=−1.5, and the value of Z=−21.5 is greater than the value of Z=−21.5. This is due to the fact that at Z=−1.5, the magnetic induction intensity is subjected to the combined action of the external magnets and the leg magnets. The simulation results show that when the distance between the external front and rear magnets is greater than 20 mm, the magnetic induction intensity between the two magnets is less than 0.17 T, and the interferences between the two magnets are small.

## 5. Test Experiments on Movement Ability of the 3D-SBIR

### 5.1. Test of the 3D-SBIR Movement on a Horizontal Plane

**Figure 8:**
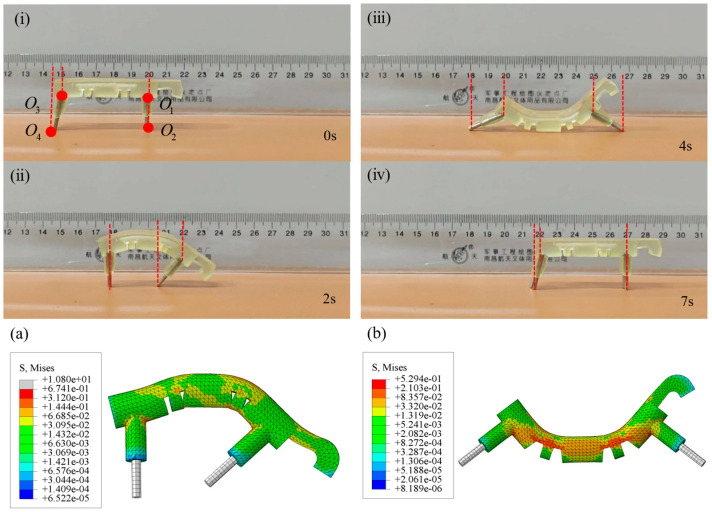
Process of the 3D-SBIR moving one step on a horizontal acrylic plate: (**i**) Initial state: Anterior legs are vertical and posterior legs are tilted backward. (**ii**) Bending deformation: Posterior legs move rightward, and belly is bending. (**iii**)Stretching deformation: Anterior legs move rightward and the belly is stretching. (**iv**) Natural state: Robot moves by a distance of 70 mm. (**a**) Stress distribution under bending deformation. (**b**) Stress distribution under stretching deformation.

**Figure 9:**
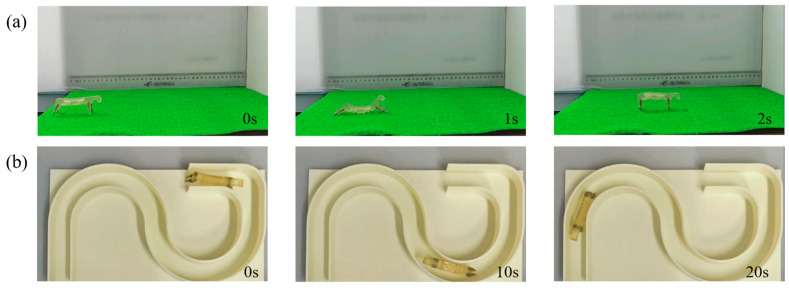
Test of the 3D-SBIR movement: (**a**) Robot moves on a grass-like plastic pad. (**b**) Robot moves through a narrow plastic runway.

To elaborate on the movement principle of the D-SBIR, the robot’s forward movement is observed on a horizontal plane. The process of the robot moving one step on a horizontal acrylic plate is shown in Figure 8. As illustrated in Figure 8i, to clearly describe the movement process of the robot, the upper endpoint of the anterior legs is labeled as $O_{1}$, the lower endpoint of the anterior legs is labeled as $O_{2}$, the upper endpoint of the posterior legs is labeled as $O_{3}$, and the lower endpoint of the posterior legs is labeled as $O_{4}$. Based on experimental results, the movement can be divided into four distinct states: initial state, bending state, stretching state, and natural state. In the initial state, shown in Figure 8ⅰ, the anterior legs of the robot are vertical and the posterior legs are tilted backward. In the bending state, shown in Figure 8ii, the posterior legs move forward, with the lower endpoints of the posterior legs shifting by about 33 mm, while the lower endpoints of the anterior legs remain motionless. This causes the belly of the robot to bend upward. The stress distribution of the robot during bending deformation is shown in Figure 8a, and the maximum pressure stress is mainly concentrated in the legs, with a maximum value of 0.67 MPa. In the stretching state, shown in Figure 8iii, the anterior legs move forward, and the lower endpoints of the anterior legs move forward by about 60 mm, whereas the lower endpoints of the posterior legs stay in place. The belly forms a downward bend, and the robot undergoes stretching deformation. The stress distribution of the robot during stretching deformation is shown in Figure 8b, and the maximum tensile stress is mainly concentrated in the legs and belly, with a maximum value of 0.53 MPa. In the natural state of the robot, shown in Figure 8ⅳ, the posterior legs move forward, with the lower endpoints of the posterior legs shifting by about 35 mm, and the lower endpoints of the anterior legs remain in place, causing the robot to return to the natural state. This completes a full motion cycle, with the robot moving about 70 mm forward. Additionally, the robot is tested on a grass-like plastic pad as shown in Figure 9a and on a plastic track with a width 0.3 times the robot’s body length, as shown in Figure 9b. Videos of the experiment are available in the Supplementary Information, and the experimental results prove that the robot exhibits excellent mobility.

### 5.2. Test of Climbing Obstacles for the 3D-SBIR

**Figure 10:**
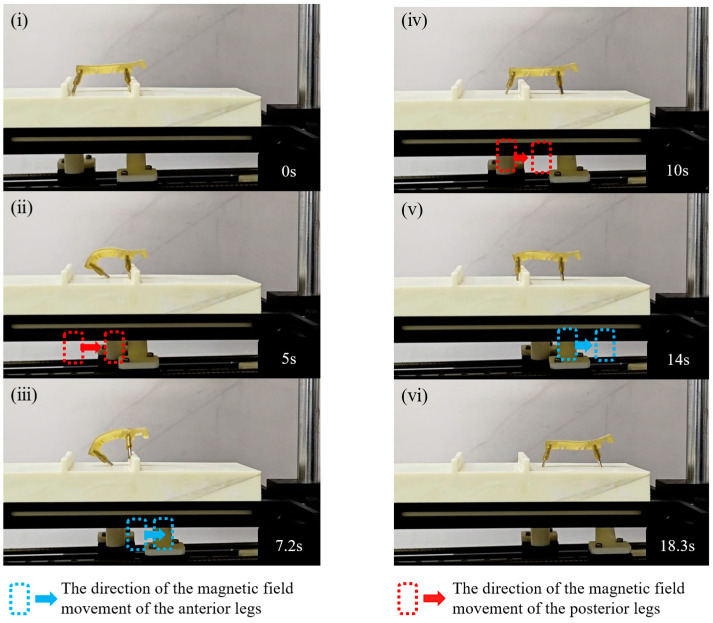
Experiment of the 3D-SBIR climbing obstacles: (**i**) Initial state of climbing obstacles. (**ii**) State of potential energy accumulation for climbing obstacles. (**iii**) State of potential energy release for climbing obstacles. (**iv**) Mobile state of climbing obstacles. (**v**) State of passive deformation of climbing obstacles. (**vi**) Natural state of climbing obstacles.

The 3D-SBIR has a soft body structure, allowing it to adapt well to its environment. In this study, this advantage is leveraged to assist the robot quickly in climbing over obstacles by utilizing contact deformation between the robot and the obstacles during the climbing process. As shown in Figure 10ⅰ, in the initial state of climbing obstacles, there are obstacles on both the anterior and posterior legs of the robot. In Figure 10ii, during the potential energy accumulation phase, the magnetic field of the anterior legs remains stationary, while the magnetic field of the posterior legs moves forward, causing the belly of the robot to bend and accumulate elastic potential energy. In Figure 10iii, during the potential energy release phase, the magnetic field of the anterior legs moves forward and the magnetic field of the posterior legs remains stationary. As the magnetic field of the anterior legs moves, the attraction force of the magnetic field of the anterior legs decreases, and the robot successfully jumps up to climb over the obstacle with the help of the elastic potential energy accumulated in the belly. In Figure 10iv, in the mobile state of climbing obstacles, after the anterior legs cross the obstacle, the posterior legs move to the front of the obstacle. In Figure 10v, during the state of passive deformation while climbing obstacles, the magnetic field of the posterior legs remains stationary, the magnetic field of the anterior legs moves forward, and the posterior legs are hindered by the obstacle, causing the belly to undergo stretching deformation. Finally, in Figure 10vi, in the natural state of climbing obstacles, the magnetic field of the anterior legs continues to move forward, and the robot climbs the obstacle successfully with the help of the pulling force of the belly.

**Figure 11:**
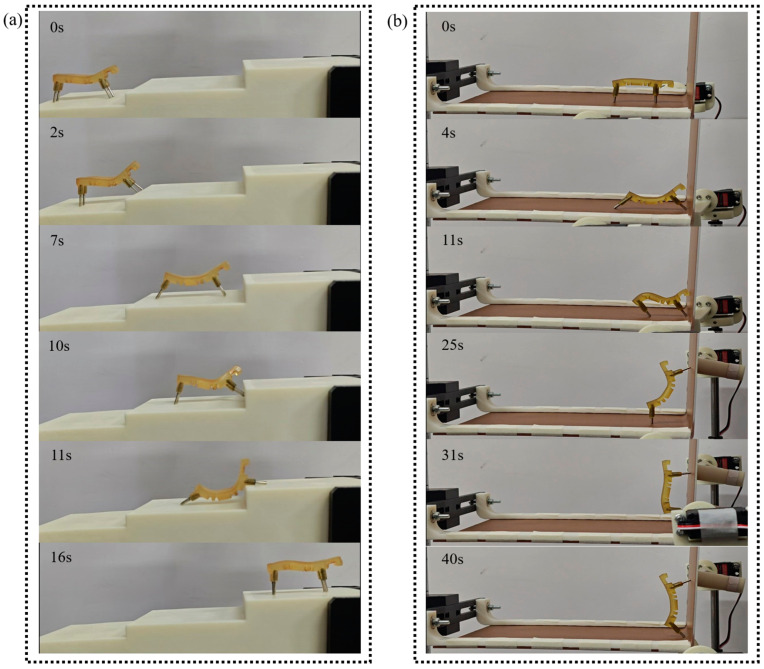
Test of motion ability in complex environments: (**a**) Climbing steps of 16mm in height. (**b**) Climbing vertical surfaces.

Experiments on climbing steps and climbing vertical planes are conducted to further validate the climbing ability of the 3D-SBIR. As shown in Figure 11a, the process of the robot climbing steps is similar to that of crossing obstacles, with the deformation of the belly assisting in the accumulation of elastic potential energy for climbing. Using a bending deformation of the belly to make the anterior legs climb steps and a stretching deformation of the belly to make the posterior legs climb steps. The 3D-SBIR is assisted by the bending deformation of its head when climbing obstacles that are significantly larger than its own height (e.g., vertical walls). The head of the robot contacts the vertical surface as shown in Figure 11b. The magnetic field of the anterior legs moves forward, causing the head to bend and deform, fitting tightly against the vertical plane. The magnetic field of anterior legs moves upward, and the robot is guided upward by the head. The magnetic field of the anterior legs moves upward, followed by the magnetic field of the posterior legs moving forward, enabling the robot to successfully climb up the vertical surface.

## 6. Conclusions

To address the problems of existing soft bionic inchworm robots, such as the complex production process and the influence of cables on the movement, this study proposes a 3D-printed soft bionic inchworm robot powered by magnetic force. First, the robot body is made directly by light-curing printing technology, and the NdFeB magnet is directly embedded in the gaps of the body as the power source. This eliminates the necessity for intricate production steps and is powered by magnetic drive, allowing cable-free operation. Additionally, the robot is capable of multimodal movement. Next, the material composition model and the bending deformation model of the robot are developed based on the Yeoh model. To validate the accuracy of the above models, simulation analysis and bending deformation experiments are conducted. The simulations and experimental results confirm the suitability of the models for the 3D-SBIR. At the same time, we discuss the main factors that influence the size of the magnetic force. With a divider thickness of 5 mm or thinner, two external magnets can provide a magnetic force of at least 0.16 N in the horizontal direction and 0.29 N in the vertical direction. Finally, unlike traditional SBIR, the 3D-SBIR utilizes its soft body structure to passively climb obstacles by making direct contact with them. As a result, the robot does not require a complex control and sensing system to have excellent obstacle-climbing capabilities. It is capable of climbing obstacles that are 0.5 times its body height with ease. The robot demonstrates multimodal movement capabilities, including the ability to traverse rough mats and narrow curved runways that are only 0.3 times its own length in width, climb steps that are 0.8 times its height, and transition from horizontal to vertical surfaces. In summary, the 3D-SBIR features a simple production process, cable-free actuation, and multimodal motion capabilities.

## Figures and Tables

**Table 1:** Comparison of the 3D-SBIR with previous inchworm-like robots.

Ref	Actuation	Cable-Free	Product Process	Movement Capability
[4]	Voltage actuation	No	Complex	Climbing pipes
[5]	Voltage actuation	No	Complex	Moving on a horizontal surface
[6]	Pneumatic actuation	No	Complex	Climbing pipes and slopes
[11]	Pneumatic actuation	No	Simple	Moving in two-dimensional space
[15]	Magnetic actuation	Yes	Simple	Moving on a horizontal surface
[16]	Magnetic actuation	Yes	Simple	Moving inside the pipeline
[17]	Magnetic actuation	Yes	Simple	Moving inside the pipeline
[22]	Magnetic actuation	Yes	Simple	Moving on a horizontal surface
This work	Magnetic actuation	Yes	Simple	Moving on a horizontal surface, climbing obstacles, steps and transitions from horizontal to vertical surfaces

**Table 2:** Design parameters of the flexible body.

Part	Parameter Name	Symbol	Value
Belly	Cavity diameter	$D_{1}$	5.0 mm
	Cavity length	$L_{1}$	30.0 mm
	Gap size	$\delta_{1}$	1.5 mm
	Gap distance	$\delta_{2}$	3.0 mm
Legs	Backward tilt angle	$\varphi_{1}$	20°
	Outward tilt angle	$\varphi_{2}$	10°
	Outside diameter	$D_{2}$	5.0 mm
	Inside diameter	$d_{1}$	2.2 mm
	Height	$H_{1}$	10.0 mm

**Table 3:** Changes in forces on the legs.

Figure	Eternal Magnetic Field Movement	Changing Trend of the Forces					
Figure 2g	Forward movement of the magnetic field of the anterior legs	$F_{N1}$ ↓	$F_{X1}$ ↑	$F_{Z1}$ ↓	$G_{1}$ ━	$F_{1}$ ━	$F_{2}$ ━
Figure 2i	The magnetic field of the anterior legs continues to move forward	$F_{N2}$ **↓**	$F_{X2}$ ━	$F_{Z2}$ ━	$G_{2}$ ━	$F_{3}$ ━	$F_{4}$ **↑**

## Data Availability

Data are contained within the article and Supplementary Materials.

## Supplementary Materials

The following supporting information can be downloaded at: https://www.mdpi.com/article/10.3390/biomimetics10040202/s1, Video S1: The motion gait analysis of the 3D-SBIR. Video S2: Test experiments on movement ability. Pdf S1: Supplementary.
