# Supplementary material for: 3D-Printed Soft Bionic Inchworm Robot Powered by Magnetic Force
Source: Biomimetics (Basel). 2025 Mar 26;10(4):202. doi: 10.3390/biomimetics10040202 (PMC12025109; doi:10.3390/biomimetics10040202)
Supplement: Supplementary file 1 [file biomimetics-10-00202-s001.zip › biomimetics-3409860-supplementary.pdf]

Supplementary

3D-printed Soft Bionic Inchworm Robot Powered by Magnetic Force

Deli Xia [1], Luying Zhang [1], Weihang Nong [1], Qingshan Duan [2] and Jiang Ding [1,3*]

1 Guangxi Key Laboratory of Manufacturing System and Advanced Manufacturing Technology, School of Me-chanical Engineering, Guangxi University, Nanning 530004, China.

2 School of Light Industry and Food Engineering, Guangxi University, Nanning 530004, China.

3 State Key Laboratory of Featured Metal Materials and Life-cycle Safety for Composite Structures, Guangxi University, Nanning 530004, China.

* Correspondence: jding@gxu.edu.cn (J D)

S1 Constitutive model of the materials

The strain-strain relationship is established based on the equation of strain energy density of the Yeoh model. The function of strain energy density is generally established by the method of Green, and the relationship between the second type of Piola-Kirchhoff stress and strain energy density is as follows:

$$K = 2\frac{\partial W}{\partial C}, \tag{S1}$$

where K is the second type of Piola-Kirchhoff stress, W is strain energy density, and C is the right Cauchy-Green deformation tensor. Under isothermal conditions, the strain energy density is expressed as three invariants of the right Cauchy-Green deformation tensor,

$$W = W\left(I_1, I_2, I_3\right), \tag{S2}$$

$$\begin{cases} I_1 = \mathrm{tr}\,C = \lambda_1^2 + \lambda_2^2 + \lambda_3^2 \\ I_2 = \dfrac{1}{2}\left[\left(\mathrm{tr}\,C\right)^2 - \mathrm{tr}\,C^2\right] = \lambda_1^2\lambda_2^2 + \lambda_2^2\lambda_3^2 + \lambda_3^2\lambda_1^2, \\ I_3 = \det C = \lambda_1^2\lambda_2^2\lambda_3^2 \end{cases} \tag{S3}$$

where I_1, I_2 and I_3 represent invariants of the right Cauchy-Green deformation tensor, and λ_1, λ_2 and λ_3 respectively represent the elongation ratios in the three main directions of the material. Equations (S1) - (S3) are combined to obtain equation (S4),

$$K = 2\left[\left(\frac{\partial W}{\partial I_1} + I_1\frac{\partial W}{\partial I_2}\right)I - \frac{\partial W}{\partial I_2}C + I_3\frac{\partial W}{\partial I_3}C^{-1}\right]. \tag{S4}$$

The second type of Piola-Kirchhoff stress and the right Cauchy-Green deformation tensor represent the stress-strain relationship of the material. The equation for the strain energy density of the Yeoh model is as follows:

$$W = \sum_{i-1}^{N} C_{i0}\left(I_1 - 3\right)^i + \sum_{k-1}^{N}\frac{1}{d_k}\left(J - 1\right)^{2k}, \tag{S5}$$

where N, C_{i1} and d_k are material constants, measured from material tensile experiments. For incompressible materials ($J = 1$), it is often taken in the form of a binomial parameter:

$$W = C_{10}\left(I_1 - 3\right) + C_{20}\left(I_1 - 3\right)^2, \tag{S6}$$

where C_{10} and C_{20} are material constants. The stress-strain relationship of the material is represented by the partial derivative of the strain energy density function with respect to the elongation ratio. The relationship between the Piola-Kirchhoff stress tensor (σ_{ij}) and the Cauchy-Green deformation tensor (γ_{ij}) is as follows:

$$\sigma_{ij} = \frac{\partial W}{\partial \gamma_{ij}} = \frac{\partial W}{\partial I_1}\frac{\partial I_1}{\partial \gamma_{ij}} + \frac{\partial W}{\partial I_2}\frac{\partial I_2}{\partial \gamma_{ij}} + \frac{\partial W}{\partial I_3}\frac{\partial I_3}{\partial \gamma_{ij}}. \tag{S7}$$

The relationship between the main stress (σ_i) and the main tensile ratio (λ_i) is obtained from equations (S1), (S3) and (S7),

$$\sigma_1 = 2\lambda_1\left[\frac{\partial W}{\partial I_1} + \left(\lambda_2^2 + \lambda_3^2\right)\frac{\partial W}{\partial I_2} + \lambda_2^2\lambda_3^2\frac{\partial W}{\partial I_3}\right], \tag{S8}$$

$$\sigma_2 = 2\lambda_2\left[\frac{\partial W}{\partial I_1} + \left(\lambda_3^2 + \lambda_1^2\right)\frac{\partial W}{\partial I_2} + \lambda_3^2\lambda_1^2\frac{\partial W}{\partial I_3}\right], \tag{S9}$$

$$\sigma_3 = 2\lambda_3\left[\frac{\partial W}{\partial I_1} + \left(\lambda_1^2 + \lambda_2^2\right)\frac{\partial W}{\partial I_2} + \lambda_1^2\lambda_2^2\frac{\partial W}{\partial I_3}\right]. \tag{S10}$$

For uniaxial tensile tests,

$$\sigma_2 = \sigma_3 = 0. \tag{S11}$$

Equations (S9) - (S11) are combined to obtain equation (S12),

$$\lambda_2^2 = \lambda_3^2. \tag{S12}$$

Then equation (S8) is simplified as,

$$\sigma_1 = \frac{2}{\lambda_1}\left(\lambda_1^2 - \lambda_2^2\right)\left(\frac{\partial W}{\partial I_1} + \lambda_2^2\frac{\partial W}{\partial I_2}\right). \tag{S13}$$

For incompressible materials,

$$I_3 = \lambda_1^2\lambda_2^2\lambda_3^2 = 1. \tag{S14}$$

From equations (S12) - (S14), the relationship between the main stress (σ_1) and the main elongation ratio (λ_1) for absolutely incompressible materials is obtained:

$$\sigma_1 = \frac{2}{\lambda_1}\left(\lambda_1^2 - \frac{1}{\lambda_1}\right)\left(\frac{\partial W}{\partial I_1} + \frac{1}{\lambda_1}\frac{\partial W}{\partial I_2}\right). \tag{S15}$$

The relationship between the invariant of the deformation tensor of the material and the main elongation ratio is as follows,

$$I_1 = \lambda_1^2 + \frac{2}{\lambda_1^2}. \tag{S16}$$

Equations (S6), (S15) and (S16) are combined to obtain equation (S20),

$$\frac{\sigma_1}{2\left(\lambda_1 - \frac{1}{\lambda_1^2}\right)} = 2C_{20}\left(\lambda_1^2 + \frac{2}{\lambda_1}\right) + C_{10} - 6C_{20} \tag{S17}$$

.

S2 Energy efficiency of the 3D-SBIR movement on a horizontal plane

As shown in Supp. Figure 1, the robot achieves a translational displacement of $s = 70\text{mm}$ over an acrylic substrate within $t = 7\text{s}$, yielding an average translational speed of $v = 10\text{mm/s}$. Robotic motion is magnetically guided by external magnets, with positional control implemented via a stepper motor-driven lead screw transmission system. The system employs a T6×4-300 series linear actuator assembly manufactured by Hongke Transmission Technology, featuring the following key specifications: Stepper motor efficiency : $\eta_1 = 80\%$; Screw lead : $P = 4\text{mm}$; Transmission system efficiency : $\eta_2 = 90\%$

(1) Effective work of the robot

The robot completes one cycle of motion, mainly overcoming friction, assuming that the weight of the robot is evenly distributed over the anterior and posterior legs.

$$F_{N1} = \frac{M}{2} * \text{g+F}_{Z1}, \tag{S18}$$

where F_{N1} is the positive pressure of the anterior legs on the acrylic plate, $M = 5.9\text{g}$ (the mass of the robot), $g = 9.8\text{m/s}^2$ (the acceleration of gravity), $F_{\text{Z1}} = 0.29\text{N}$ (the magnetic force of the anterior legs in the vertical direction)

$$f_1 = \mu * F_{\text{N1}}, \tag{S19}$$

$$W_{\text{eff1}} = f_1 * \text{s}, \tag{S20}$$

where f_1 is the friction force on the anterior legs, W_{eff1} is the effective work of the anterior legs, and $\mu = 0.3$ (the coefficient of surface kinetic friction of the acrylic plate). The effective work of the posterior legs, which move forward by 70mm like the anterior legs, overcoming the friction force, is therefore known to be $W_{\text{eff2}} = W_{\text{eff1}}$. The effective work of the robot is

$$W_{\text{eff}} = W_{\text{eff2}} + W_{\text{eff1}}. \tag{S21}$$

(2) Input and output power of stepper motors

$$n_1 = v / P, \tag{S21}$$

$$w_1 = 2 * \pi * n_1, \tag{S22}$$

$$T_1 = \frac{f_1 * P}{2 * \pi * \eta_2}, \tag{S23}$$

$$P_{\text{out1}} = T_1 * w_1, \tag{S24}$$

$$P_{\text{in1}} = \frac{P_{\text{out1}}}{\eta_1}, \tag{S25}$$

where n_1 is the rotational speed of the stepper motor of the anterior legs, w_1 is the acceleration of the stepper motor of

the anterior legs, T_1 is the torque of the stepper motor of the anterior legs, P_{out1} is the output power of the stepper motor of the anterior legs, and P_{in1} is the input power of the stepper motor of the anterior legs.

Similarly, the input power of the stepper motor of the posterior legs is $P_{\text{in 2}} = P_{\text{in1}}$.

(3) Energy efficiency of the robot

$$W = P_{\text{in1}} * t + P_{\text{in2}} * t \,. \tag{S26}$$

$$\eta = \frac{W}{W_{\text{eff}}} \tag{S27}$$

where W is the total input energy and η is the energy efficiency of the robot. From equation (S18) to (S27), it can be obtained,

$$\eta = 71.95\% \,. \tag{S28}$$

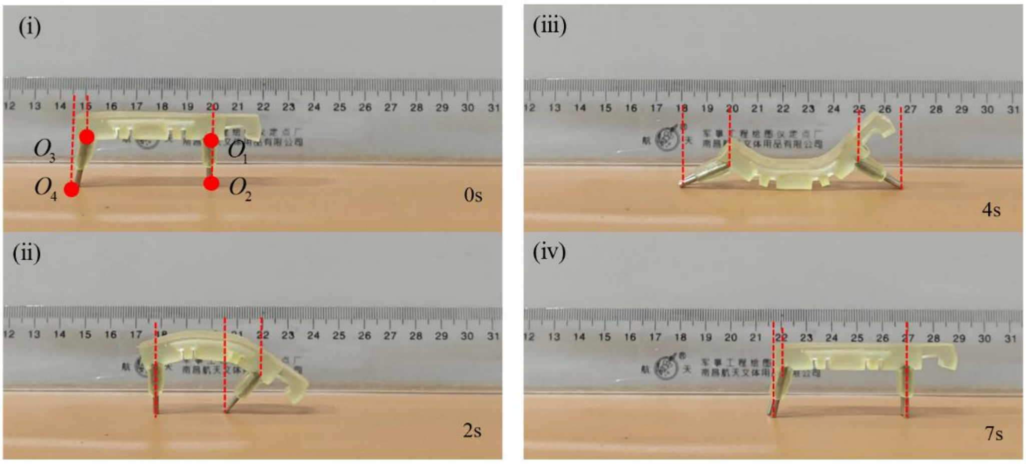

Supp. Figure 1. Process of the 3D-SBIR moving one step on a horizontal acrylic plate: **(i)** Initial state: Anterior legs are vertical and posterior legs are tilted backward. (**ii**) Bending deformation: Posterior legs move rightward, and belly is bending. (**iii**)Stretching deformation: Anterior legs move rightward and the belly is stretching. (**iv**) Natural state: Robot moves by a distance of 70 mm.
